# Immunosuppression is associated with loss of airway microbiome resilience in severe COVID-19

**DOI:** 10.1186/s40168-026-02462-1

**Published:** 2026-07-23

**Authors:** Matthew Chung, Clea R. Barnett, Lars Hunger, Imran Sulaiman, Benjamin G. Wu, Yonghua Li, Jun-Chieh J. Tsay, Shari B. Brosnahan, Cecilia J. Chung, Luis Angel, Gregg J. Silverman, James T. Morton, Leopoldo N. Segal, Elodie Ghedin

**Affiliations:** 1 Systems Genomics Section, Laboratory of Parasitic Diseases, National Institute of Allergy and Infectious Diseases, National Institutes of Health, Bethesda, MD USA; 2 Division of Pulmonary and Critical Care Medicine, New York University Grossman School of Medicine, NYU Langone Health, New York, NY USA; 3 Department of Medicine, New York University Grossman School of Medicine, NYU Langone Health, New York, NY USA; 4 Gutz Technologies, Corvina, CA USA; 5 Division of Pulmonary and Critical Care Medicine, VA New York Harbor Healthcare System, New York, NY USA; 6 Division of Rheumatology, Department of Medicine, New York University Grossman School of Medicine, NYU Langone Health, New York, NY USA; 7 Laura and Isaac Perlmutter Cancer Center, New York University Grossman School of Medicine, NYU Langone Health, New York, NY USA

**Keywords:** COVID-19, Respiratory microbiome, Metatranscriptomics, Lower airway dysbiosis, Immunosuppression

## Abstract

**Background:**

The role of the lower airway microbiome in COVID-19-associated mortality remains poorly understood despite evidence implicating secondary pathogens and non-airway commensals. The influence of prior immunosuppression on the lower airway microbiome and its contribution to mortality in mechanically ventilated patients has been largely unexplored. Given that host-microbiome interactions are dynamic, we assessed longitudinal patterns in the respiratory microbiome associated with mortality and immunosuppression status.

**Results:**

We conducted longitudinal forecasting, differential abundance, and comparative network analyses of metatranscriptomic data from 604 upper and lower airway samples collected from 243 mechanically ventilated COVID-19 patients to profile key microbial factors associated with immunosuppression and clinical outcome. SARS-CoV-2 was enriched over time in immunosuppressed patients and in both immunosuppressed and immunocompetent patients who died, highlighting the need for aggressive viral infection control early in the disease course. Using longitudinal forecasting and differential abundance analyses, we observed a progressive increase in opportunistic pathogens, such as *Staphylococcus aureus* and *Escherichia coli*, among the taxa most predictive of mortality. Concurrently, immunosuppressed individuals exhibited a significant decrease in typical airway commensals alongside a restructuring and marked destabilization of the microbial community compared with their immunocompetent counterparts.

**Conclusions:**

These findings indicate that loss of airway microbiome stability in immunosuppressed patients constitutes an independent factor contributing to increased susceptibility to opportunistic pathogens and poor clinical outcomes.

Video Abstract

**Supplementary Information:**

The online version contains supplementary material available at https://doi.org/10.1186/s40168-026-02462-1.

## Background

Respiratory infections cause significant changes in the airway microbiome. In conditions such as COVID-19 or influenza, the airway microbiome is notably different from that of the healthy state [1]. One of the main causes of mortality in COVID-19 is ARDS (acute respiratory distress syndrome), which results from a dysregulated inflammatory response to SARS-CoV-2 [2, 3]. ARDS has also been found to co-occur with bacterial pneumonia, with approximately 25% of COVID-19 patients presenting with secondary infection at the time of intubation [4–8]. In ARDS, the presence of gut- or oral-associated commensals in the lungs has been linked to lung inflammation and pneumonia [9, 10]. We previously observed a similar trend with COVID-19, in which the increased abundance of the oral commensal *Metamycoplasma salivarium* in the lower airways of intubated COVID-19 patients was associated with mortality [11].

It is also important to understand the complex role of typical airway commensals, which can become opportunistic pathogens when their abundance reaches a critical threshold following a disruption in the microbiome. For example, taxa such as *Staphylococcus*, *Ralstonia*, and *Enterococcus* spp., along with secondary bacterial pneumonia, have been identified as predictors of negative clinical outcomes in COVID-19 [9, 12]. These findings all suggest that an imbalanced lung microbiome significantly influences disease onset, progression, and prognosis.

This relationship is further complicated by the connection between the microbiome and the local immune response. SARS-CoV-2 infection triggers a robust inflammatory immune response, and the presence of other bacteria in the lower airways, even if they are common commensals in other mucosae, could exacerbate this inflammation. While corticosteroid treatment is used to reduce maladaptive inflammation in severe COVID-19 cases [13], its anti-inflammatory effects also suppress the immune system. This is further complicated in individuals who are already immunosuppressed, as a strong immune response is needed to limit and clear SARS-CoV-2 infection. Because of this, immunosuppressed individuals have an increased mortality risk compared to the healthy population [14–16], with immunosuppression being associated with higher viral loads, delayed viral clearance from the airways, and a significantly increased risk of mortality in COVID-19 [14–17].

The concept of microbiome resilience refers to the ability of a niche to withstand or recover from a perturbation [18]. This resilience is maintained through a network of microbe-microbe and host-microbe interactions. In the lung, resilience has been linked to colonization resistance, in which the local microbiome and immune system work together to control the growth of opportunistic pathogens. In respiratory diseases such as pneumonia, an impaired immune system weakens colonization resistance, leading to a higher risk of further infection [19]. Understanding how immunosuppression impacts the dynamics and composition of the lower airway microbiome is crucial for predicting outcomes and managing mortality risk in these vulnerable individuals.

In this study, we build upon our previous cross-sectional study [11] with a longitudinal assessment of the lower-airway microbiomes in mechanically ventilated COVID-19 patients, using samples collected over a period of 3–4 weeks. Using this longitudinal dataset, we employ data forecasting approaches, differential abundance analyses, and network stability analyses to identify specific factors that contribute to or are linked with immunosuppression and mortality, including the progressive enrichment of the lower airways with upper-airway commensals and opportunistic pathogens.

## Methods

### Subjects

The cohort included 243 subjects 18 years or older admitted to the New York University (NYU) Langone Health intensive care units (ICUs) from March 11, 2020, to March 30, 2022, with all patients having met ARDS criteria, a nasal swab-confirmed diagnosis of SARS-CoV-2 using RT-PCR, and respiratory failure requiring invasive mechanical ventilation. Lower airway samples were obtained during clinically indicated bronchoscopies or tracheal aspirates performed for airway clearance and/or percutaneous tracheostomy placement. Background control samples were obtained by passing saline through the bronchoscope prior to bronchoscopy, as previously described [20]. All patients or their legal representatives consented to participate in the study via the NYU IRB-approved protocol (IRB #s16-00122/01598), with signed consent obtained either upon patient recovery or from a legally authorized representative for deceased individuals. Demographic and clinical data were collected from patients, and all downstream analyses used de-identified data. The study protocol was approved by the NYU Institutional Review Board.

### Sample collection, RNA isolation, and sequencing

RNA was isolated using zymoBIOMICS™ DNA/RNA Miniprep Kit (Cat: R2002) as per the manufacturer’s instructions. RNA quality was assessed using the BioAnalyzer 2100 (Agilent). Library preparation was done using the automated Nugen Ovation Trio Low Input RNA method with 3ng total RNA input for each sample. After 6 amplification cycles, samples were sequenced using Illumina NovaSeq 6000 S4 200 cycle flow cells and 100 bp paired-end sequencing (Illumina).

### Upstream processing

Raw paired-end reads were processed using fastp v0.23.4 [21] to trim adaptor sequences and remove PCR duplicates. Host sequences were filtered by removing any reads that mapped to the GrCh38 human genome using Bowtie v2.5.1 [22]. The processed reads were assigned taxonomy with Kraken v2.1.2 [23], using human, bacteria, fungi, archaea, and viral RefSeq databases along with the EuPathDB46 database. Additional reads identified as human were removed using KrakenTools v1.2 [24]. Abundances of taxa were statistically estimated using Bracken v2.8 [24]. Reads assigned to *Homo sapiens*, synthetic constructs, and protozoa were removed from quantification and samples with ≥ 10,000 taxa counts were used for downstream analyses. As our bronchoalveolar lavage (BAL) samples were taken predominantly in our first wave of sequencing while tracheal aspirates (TA) were taken in later waves, lower airway sample type and sequence batch were largely confounded in this dataset, making it difficult to use sample type as an independent covariate. Therefore, we applied run-level batch correction using ConQuR v2.0 [25] and empirically assessed whether the two sample types remained compositionally distinct. Principal coordinates analyses (PCoAs) generated using Bray–Curtis dissimilarity indices were calculated from the normalized counts using vegan v2.6–6.1 [https://CRAN.R-project.org/package=vegan] and showed the BAL and TA samples to cluster together, indicating the compositional differences between the two sample types were largely due to sequencing batch rather than intrinsic differences. Contaminant taxa were flagged using decontam v1.24 [26] run with prevalence using taxa counts as the library size, background samples as the negative, and a threshold ≥ 0.5.

For antimicrobial resistance analyses, human-depleted reads were quantified using CoverM v0.7.0 [27] against the MEGARes v2.0 database [28]. Counts assigned to genes that conditionally confer resistance based on single nucleotide polymorphisms (SNPs) were removed. Counts were batch-corrected using ConQuR v2.0 [25] and combined based on conferred resistance category for downstream analyses.

### Diversity and differential abundance analyses

Species abundances were normalized by first estimating size factors using the median ratio method, followed by variance stabilizing transformation with DESeq2 v1.44.0 [29]. Alpha diversity analyses were conducted by calculating Shannon diversity indices and comparing groups for significance (*p* < 0.05) using the Kruskal–Wallis test. Bray–Curtis dissimilarity indices were calculated from the normalized counts using vegan v2.6–6.1 [https://CRAN.R-project.org/package=vegan]. We conducted PCoAs using the Bray–Curtis dissimilarity indices and performed ADONIS PERMANOVA tests to assess clustering significance between different covariates.

Differential abundance analyses for taxa, functional categories, and antimicrobial resistance were performed using edgeR v4.2.0 [30] with TMM normalization applied to a generalized linear model. Low-abundance taxa with < 1% prevalence and < 0.003% average relative abundance were removed in downstream analyses. A quasi-likelihood *F* test was used to identify significant features (FDR < 0.05). For each week, a separate differential abundance analysis was conducted comparing either immunosuppression status or outcome, using only the earliest sample during each week for an individual.

Comparative networks were constructed using NetCoMi v1.1 [31] using the abundances of the top 100 taxa across each week (see online supplement for details).

### DVAE and logistic regression analysis

A dynamical variational autoencoder (DVAE) model was created by combining variational autoencoders (VAEs) with a differentiable Kalman Filter to forecast data across our longitudinal dataset. In brief, each omics dataset is first compressed into a low-dimensional representation that retains its key biological features. These representations are then linked across timepoints using the Kalman Filter to model how host and microbial profiles co-evolve, enabling the prediction of missing measurements to forecast data. The forecasted data were evaluated using a logistic regression model to identify discriminative balance (DB) values for each taxa, determining whether they contributed more to a positive versus negative clinical outcome (see online supplement for details).

## Results

### Study cohort

In a previous study, we focused on a group of COVID-19 patients admitted to the ICUs at NYU Langone Health in New York (Long Island and Manhattan) during the first wave of the pandemic, from March 3rd to June 18th, 2020 [11]. While that initial study allowed us to associate lower airway metatranscriptome profiles with clinical covariates, it did not capture the impact of SARS-CoV-2 infection on the microbiome over time.

**Fig. 1:**
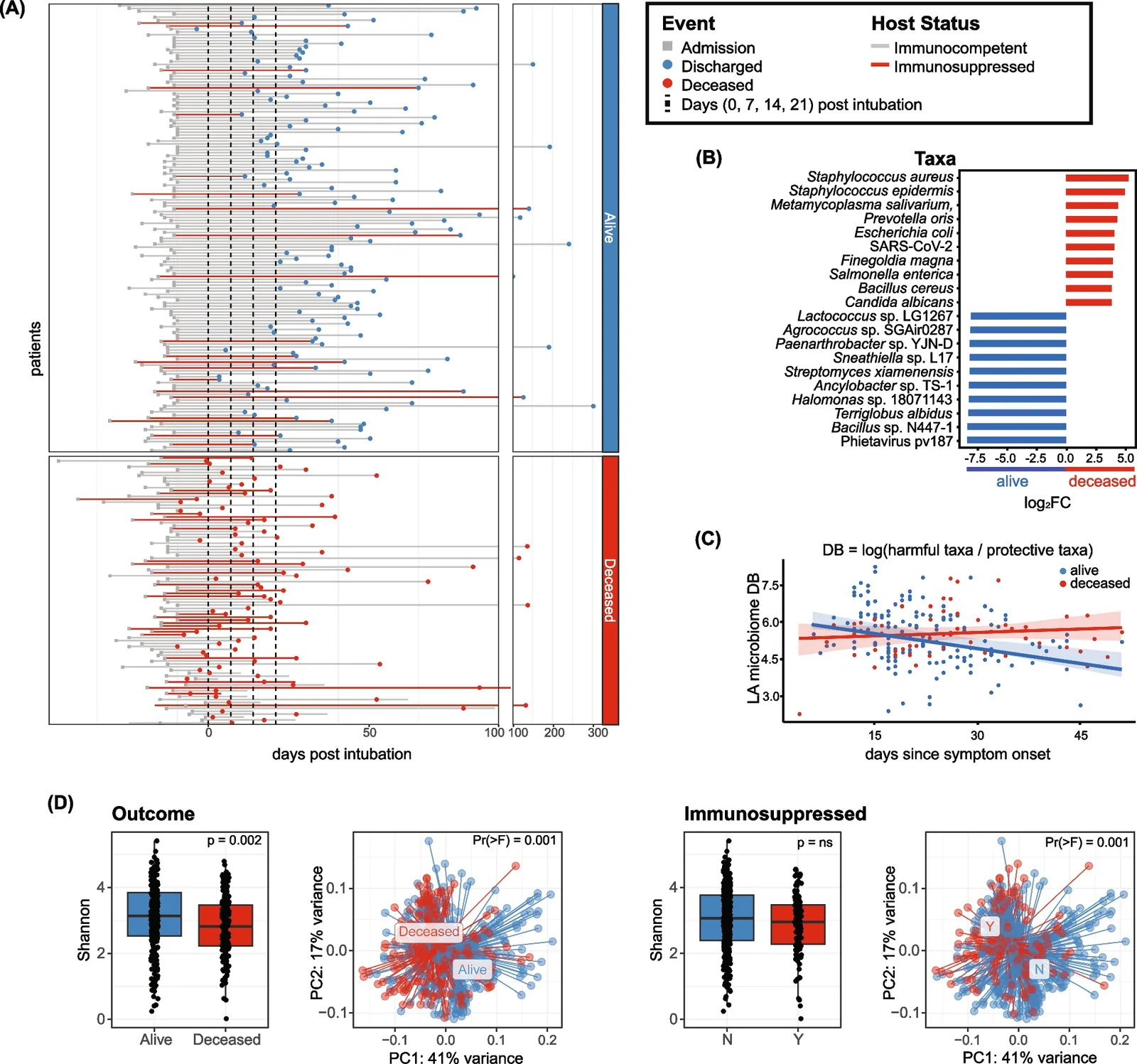
Longitudinal cohort design and airway microbiome dynamics associated with clinical outcome and immunosuppression. **A** Dot-plot illustrating longitudinal sampling of mechanically ventilated COVID-19 patients from admission (grey squares) to discharge (blue dots) or death (red dots). Each line represents an individual patient, colored by immunosuppression status (red, immunosuppressed; grey, immunocompetent). Vertical dashed lines indicate days post-intubation (0, 7, 14, and 21). **B** Taxa in bronchoalveolar lavage (BAL) samples associated with clinical outcome, identified using a deep variational autoencoder (DVAE) model. Shown are the ten taxa with the highest and lowest log-fold changes, corresponding to enrichment in deceased and surviving patients, respectively. **C** Discriminative balance (DB) of lower airway microbial communities, calculated from taxa associated with clinical outcome. Higher values indicate enrichment of taxa associated with mortality. Longitudinal trajectories are shown for individual samples, stratified by outcome. **D** Shannon diversity indices and Bray–Curtis distances were calculated and compared between clinical outcome (alive, deceased), and immunosuppression status. Kruskal–Wallis tests were used to identify significance (*p* < 0.05) between groups for Shannon diversity indices. Bray–Curtis distances were plotted on a PCoA and PERMANOVA tests were used to identify significant differences (Pr(> F) < 0.05) between groups

To address this, we collected additional samples longitudinally through March 30, 2022 from 243 mechanically ventilated COVID-19 patients, including 50 who were immunosuppressed and 91 who died (Fig. 1A; Supplementary Table S1A). This cohort includes 445 lower-airway samples (LA) and 159 upper-airway samples (UA), with 1–6 samples of each type per individual (Table 1; Supplementary Fig. S1A, 1B). Additionally, while the LA samples collected in the previous study were all from bronchoalveolar lavages (BAL), we obtained additional lower-airway samples by including tracheal aspirates (TA), which are less invasive and thus collected more regularly. UA samples were collected using supraglottic suctioning.

**Table 1:** Cohort and sample overview

	Samples (*N* = 604)		
Variable	BAL	TA	UA
*N*	210	235	159
Individuals (*N* = 243)	169	99	107
Age	62 (49–64)	63 (52–70.5)	62 (51–70)
BMI	28 (24–32)	30 (26–35)	28 (24–33)
Duration of mechanical ventilation (days)	31 (22.75–46)	26 (12–46)	30 (20–43.8)
ddPCR copies per µL	2173 (260–11,140)	3606.5 (1,210.3–54,150)	314,666.5 (32,799.8–3,158,000)
Antibiotic exposure score	71 (49–109.6)	71 (35.5–177.5)	71 (35.5–107.5)
Samples taken			
* 1 week post-intubation*	56 (26.7%)	100 (42.6%)	57 (35.8%)
* 2 weeks post-intubation*	85 (40.5%)	54 (23.0%)	48 (30.2%)
* 3* + *weeks post-intubation*	69 (32.9%)	81 (34.5%)	54 (34.0%)
Samples per individual	1.2 (1–6)	2.4 (1–6)	1.5 (1–6)
Deceased (*N* = 91)	53 (25.2%)	121 (51.5%)	68 (42.8%)
Immunosuppressed (*N* = 50)	32 (15.2%)	67 (28.5%)	42 (26.4%)
Sex (male; *N* = 174)	164 (78.1%)	147 (62.6%)	105 (66.0%)
Race			
* Caucasian* (*N* = 117)	94 (44.8%)	107 (45.5%)	78 (49.1%)
* Black/African American* (*N* = 25)	13 (6.2%)	33 (14.0%)	19 (11.9%)
* Asian* (*N* = 27)	16 (7.6%)	37 (15.7%)	17 (10.7%)
* Unknown/Other* (*N* = 74)	87 (41.4%)	58 (24.7%)	45 (28.3%)
Ethnicity			
* Hispanic or Latino* (*N* = 52)	58 (27.6%)	50 (21.3%)	38 (23.9%)
Smoking status			
* Ever* (*N* = 70)	51 (24.3%)	78 (33.2%)	45 (28.3%)
* Never* (*N* = 173)	159 (75.7%)	157 (66.8%)	114 (71.7%)
COVID-19 vaccinated (*N* = 21)	5 (2.4%)	46 (19.6%)	22 (13.8%)
ECMO (*N* = 46)	70 (33.3%)	61 (26.0%)	24 (15.1%)

Patients were considered immunosuppressed if they had a history of HIV/AIDS, metastatic solid malignancy, or active hematologic cancer. This includes individuals undergoing active cancer treatment, those with a prior stem cell or solid-organ transplant, and people with chronic pulmonary diseases such as interstitial lung disease or sarcoidosis. Other conditions indicating immunosuppression included dialysis, a prior splenectomy, rheumatoid arthritis, or other autoimmune diseases (Supplementary Table S1B). Additionally, patients were considered immunosuppressed if they were on immunosuppressive drugs (Supplementary Table S1C), including a prednisone dose of ≥ 10 mg-equivalents per day for at least 2 weeks within 2 weeks of symptom onset.

For each sample, we calculated taxa abundances to profile the microbiome. Bray–Curtis dissimilarity indices were calculated from normalized species abundances and visualized using PCoA. While the PCoA showed distinct clusters for the UA and background samples, there was significant overlap between BAL and TA samples, indicating a high degree of similarity between the two types of LA samples (Supplementary Table S1D; Supplementary Fig. S1C).

### Temporal modeling identifies key features predictive of clinical outcome

To better understand disease progression in our cohort, we needed to address a key challenge: since airway sampling was driven by clinical need rather than a fixed protocol, most patients had measurements at irregular timepoints, making it difficult to track how microbial communities changed over time. To fill these gaps, we developed a forecasting model that combines five available data streams to predict missing measurements: the BAL microbiome and TA microbiome from this study, along with the BAL host transcriptome, TA host transcriptome, and blood transcriptome from the same cohort described in our previous study [11] (Supplementary Fig. 2 A). Due to the sparse sampling of the UA, we excluded it from the forecasting model.

This forecasting approach, called a dynamical variational autoencoder (DVAE), works in two stages. First, each data type is compressed into a compact summary that captures its essential biological variation, similar to how a principal component analysis (PCA) reduces high-dimensional data to its dominant axes, but the DVAE has the added ability to capture nonlinear relationships. Second, these summaries are linked across time using a Kalman filter [32], a forecasting technique widely used in engineering and signal processing, that learns how the compressed representations of each data type co-evolve and uses observed timepoints to predict unobserved ones. Because the model learns the temporal relationships between host and microbial signals simultaneously, it can leverage information from one data type (e.g., a blood transcriptome sample collected at week 2) to improve predictions for another (e.g., a missing microbiome measurement at that same timepoint). By using the forecasted taxa abundances and performing statistical analyses, we aimed to identify key differences between patient outcomes.

To evaluate the DVAE model, we divided the data into 80/10/10 training, validation, and test sets, and assessed its ability to forecast either host gene abundances in the host transcriptomic datasets or microbial taxa abundances in the microbiome datasets. In the validation cohort, we observed high concordance between the predicted and observed abundances in host blood transcriptomics (*r* = 0.86), BAL host transcriptomics (*r* = 0.77), and BAL microbiome (*r* = 0.51) (Supplementary Fig. S2B). The forecasting accuracy for the TA microbiome (*r* = 0.48) was similar to the BAL microbiome, while the forecasting accuracy of the TA host transcriptomics was low (*r* = 0.19). The variation in forecasting accuracy across datasets shows higher concordance for data streams with more consistent temporal structure (blood and BAL host transcriptomics) and lower concordance for compositionally complex data (microbiome) or sparsely sampled data (TA host transcriptomics). The moderate per-species correlations contrast with the strong overall aggregate signal: downstream classifiers built on DVAE-imputed data achieved an AUC of 0.90 for predicting mortality from BAL taxa alone (Supplementary Fig. S2C), substantially outperforming matched random forest (RF) classifiers trained on the same imputed features and evaluated on the same held-out patients (AUC = 0.50). The RF’s poor performance likely reflects the advantage of the discriminative balance framework, which leverages biologically informed log-ratios of harmful-to-protective taxa, over a model that treats each taxon independently without considering compositional structure. These results confirm that the forecasted profiles capture biologically meaningful variation despite imperfect reconstruction of individual species.

Using the DVAE model, we compared individuals who survived with those who died. We used dynamic time warping to assess the similarity between patients based on their time series. PERMANOVA testing revealed a statistically significant difference between outcomes (*p* < 0.01). We then performed a longitudinal differential abundance analysis on the forecasted data to identify features that differentiated patients by their outcome, using log-fold changes calculated from imputed abundances with the DVAE. Among the top ten taxa from the BAL microbiome most significantly associated with mortality were *Staphylococcus aureus*, *Staphylococcus epidermidis*, *Escherichia coli*, SARS-CoV-2, and *Candida albicans* (Fig. 1B). This supports ongoing observations that SARS-CoV-2 infection is associated with an increased risk of potential pathogens colonizing the lower airways [33].

A discriminative balance (DB) analysis was also performed in which two groups of taxa were selected: one with the strongest association with death (harmful taxa) and another with the weakest association (potentially protective taxa). A single DB ratio was calculated for each LA sample (BAL and TA microbiome) by taking the log-ratio of these two taxonomic groups. Using a linear mixed effects analysis, we assessed differences between survivors and deceased patients while controlling for immunosuppressed status, age, sex, and BMI. To evaluate the generalizability of the logistic regression model, we assessed its prediction accuracy on a holdout test cohort (*n* = 34). We found that DB progression in the LA samples was significantly different over time between survivors and deceased patients (Fig. 1C). Patients who eventually died showed a slight increase in their DB over time, which could potentially reflect their inability to decrease the load of harmful taxa. In contrast, patients who eventually recovered experienced a significant decrease in their DB over time, likely corresponding to a reduction in pathogenic microbes and an increase in commensal microbes.

In addition, we identified the top host genes associated with mortality. Of these, several mitochondrial genes were among the top features predicting mortality in the BAL classifier (Supplementary Fig. S2D). Specifically, mitochondrial ribosomal RNAs (RNR2, RNR1), NADH dehydrogenase subunits (ND2, ND4, ND5), and cytochrome c oxidase subunits (COX1, COX3) were all strongly associated with the deceased group, reflecting increased cellular damage and death in the airways of patients with fatal outcomes.

### Airway commensals are enriched in survivors and immunocompetent patients

To further explore this association between taxa and outcomes, we aimed to assess the effect of immunosuppression on that dynamic. Given that the BAL and TA microbiomes were similar in both the PCoA and temporal model forecasting accuracies, we continued to combine the BAL and TA samples to represent the LA in downstream analyses. As the LA microbiome (Supplementary Fig. S3A) provided greater sensitivity and informative value compared to the UA (Supplementary Fig. S3B) in the context of ventilated patients with COVID-19, we focused primarily on the lower airways for further downstream analyses.

Since clinical outcome and immunosuppression were two factors significantly associated with separation of LA samples (Fig. 1D; Supplementary Fig. S3–S5; Supplementary Table S1D), we performed differential enrichment analyses on the LA sample subsets for weeks 1, 2, and ≥ 3 post-intubation. We filtered out low-abundance taxa with < 0.1% average relative abundance and < 1% prevalence across the cohort. In the LA, the retained taxa accounted for an average of 85.51% (IQR 80.71–93.02%) of taxa counts whereas in the UA, the retained taxa accounted for an average of 88.46% (IQR 83.72–93.66%), indicating that the filtered taxa collectively represent a small fraction of the total microbial signal. Other clinical metadata were also found to be associated with differences in LA microbiome composition and are further discussed in the online supplement.

**Fig. 2:**
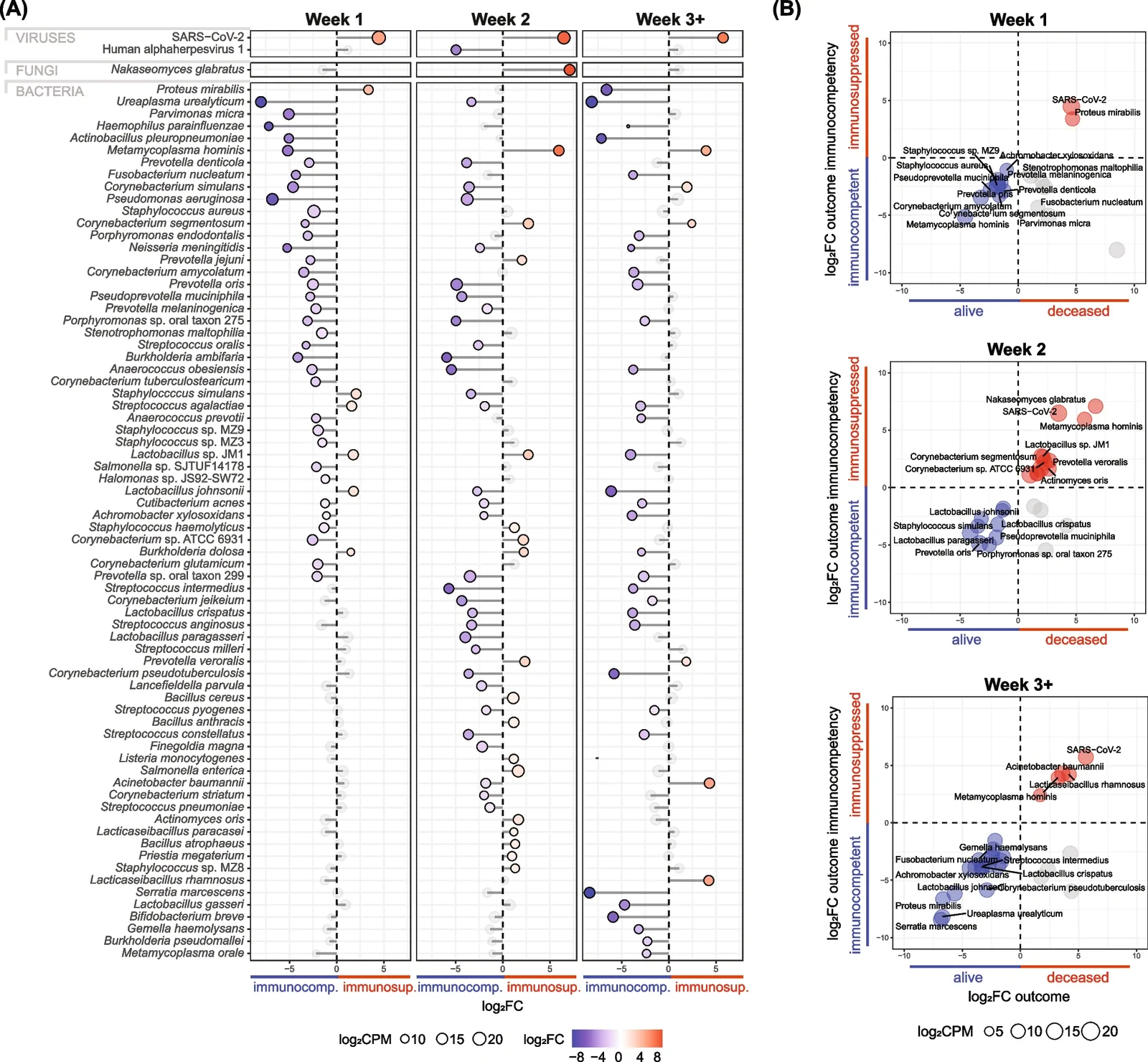
Differential enrichment and abundance of taxa according to immunosuppression status and clinical outcomes. **A** Fold-change bubble plots showing differentially enriched taxa in the lower airways by immunosuppression status. For each week, differential expression analyses identified significantly enriched taxa (FDR < 0.05) in immunosuppressed (red) versus immunocompetent (blue) patients. Only significant taxa are colored. Bubble color reflects log-fold change (logFC), and size reflects log counts per million (log_2_CPM). **B** Differentially abundant taxa across timepoints (weeks 1, 2, and ≥ 3) in lower airway samples. Log-fold changes associated with clinical outcome (*x*-axis) and immunosuppression status (*y*-axis) are shown for significant taxa (FDR < 0.05), with bubble size proportional to log_2_CPM. Colors indicate direction of enrichment (red, deceased and immunosuppressed; blue, alive and immunocompetent; grey, mixed associations)

Comparing the immunosuppressed to the immunocompetent groups, we found more differentially abundant taxa in the immunocompetent group across all three sample subsets by week (Supplementary Fig. S6A). In the immunosuppressed group, only SARS-CoV-2 was consistently differentially enriched in all 3 weeks (Fig. 2A). Among other taxa identified as differentially enriched, *Burkholderia dolosa*, a species often associated with cystic fibrosis and frequently identified in the airways of immunosuppressed individuals [34, 35], and *Lactobacillus* sp. JM1, a bacterial species typically linked to the oral cavity and gastrointestinal tract [36–38], were both enriched in the LA of the immunosuppressed during weeks 1 and 2 (Fig. 2A).

In immunosuppressed individuals, we also identified three taxa enriched in weeks 2 and ≥ 3: *Metamycoplasma hominis*, *Prevotella veroralis*, and *Corynebacterium segmentosum*. While *M. hominis* is typically found in the genital tract [39, 40], it has been linked to extragenital infections and is a common colonizer in chronic lung disease [41, 42]. Both *Prevotella* and *Corynebacterium* spp. are also typical upper-airway commensals but have been reported to be more abundant in individuals with COVID-19 [43–45]. Additionally, *Corynebacterium* spp. are usually found in the upper airways, specifically the nasal cavity and nasopharynx [38]. This increased presence of non-lower-airway commensals could indicate microbiome dysbiosis in the lower airways of immunosuppressed patients. Furthermore, in immunosuppressed individuals, we observed the highest number of differentially enriched taxa at week 2 compared to weeks 1 and ≥ 3.

In immunocompetent individuals, while more taxa are differentially enriched overall, the number remained stable across all ≥ 3 weeks (32 to 35; Supplementary Fig. S6A). Most of these taxa are typical airway microbes, including several *Streptococcus* spp. [38, 46]. We also observe other *Prevotella* spp. significantly enriched across all timepoints, including *P. oris* and *Prevotella* sp. oral taxon 299 (Fig. 2A) [47]. The increased prevalence of typical LA commensals in immunocompetent individuals suggests greater stability in the LA microbiome compared to immunosuppressed patients. We also observed several *Corynebacterium* spp. enriched in the LA of the immunocompetent, despite being typically found in the upper airways [38, 48]. Some *Corynebacterium* spp. have shown protective effects against opportunistic bacterial pathogens, such as *S. pneumoniae* and *S. aureus* [49].

As antibiotic or corticosteroid exposure could confound these findings, we recorded antibiotics and steroids administered prior to sampling for each patient. To evaluate antibiotic exposure, we used an antibiotic exposure score (AES) that reflects the breadth of antibiotic spectrum used. We repeated the differential abundance analysis incorporating AES as a covariate by splitting samples by the median AES score (71), and found the results to be highly concordant with those derived using immunosuppression status alone, with 31 of 42, 46 of 51, and 31 of 36 taxa remaining differentially abundant between groups across weeks 1, 2, and ≥ 3, respectively (Supplementary Fig. S7A and S7B). We performed the same analysis but instead incorporating corticosteroid use prior to each sample collection date as a covariate, again yielding highly concordant results, with 30 of 42, 45 of 51, and 34 of 36 differentially abundant taxa shared across weeks 1, 2, and ≥ 3, respectively (Supplementary Fig. S7A and S7C). This indicates that neither antibiotic nor corticosteroid exposure substantially influences the observed differences in LA microbiota composition between immunosuppressed and immunocompetent patients.

We performed the same differential enrichment analysis focusing on taxa linked to clinical outcomes (Supplementary Fig. S6A). Across all 3 weeks, SARS-CoV-2, *Bacillus cereus*, *Actinomyces oris*, and the fungi *Fusarium oxysporum* were all enriched in the deceased group (Supplementary Fig. S6B). The taxa differentially enriched in week 3 and above in the lower airways of the deceased individuals included several *Bacillus* and *Staphylococcus* spp., bacterial genera known to be associated with opportunistic airway infections [50–52]. Additionally, an increased abundance of *B. cereus* in the upper airways has previously been linked to increased COVID-19-associated mortality [53].

There were 34 significantly differentially enriched taxa linked to the survivor group in week ≥ 3 compared to 15 in weeks 1 and 2 individually (Supplementary Fig. S6A), including *Prevotella* and *Streptococcus* spp. (Supplementary Fig. S6B). This mirrors findings in immunocompetent individuals, where a greater abundance of airway commensals is associated with recovery. We also find *Corynebacterium* spp. to be associated with the survivor group, further indicating a potential protective effect of certain *Corynebacterium* spp. in the LA. Additionally, the number of differentially enriched taxa identified between alive and deceased patients was less pronounced than that seen between immunocompetent and immunosuppressed groups.

We visualized the results of both differential expression analyses for immunocompetency status and clinical outcome by plotting them together (Fig. 2B). During the first week, only SARS-CoV-2 and *Proteus mirabilis* were enriched in immunosuppressed individuals who eventually died, while *Corynebacterium* spp. and *Prevotella* spp. were found in the immunocompetent survivors. We observed enrichment of several *Staphylococcus* spp. and *Metamycoplasma hominis* in the deceased group in later weeks, despite being associated with the survivor group early on.

In the second week, SARS-CoV-2 is again linked to the deceased immunosuppressed, along with *M. hominis*, *C. segmentosum*, and the fungus *Nakaseomyces glabratus*. While *N. glabratus* has previously been associated with respiratory infections in immunosuppressed individuals [54, 55], its clinical significance in the airway remains unclear and could serve as a biomarker for broad-spectrum antibiotic use leading to commensal *Candida* overgrowth [56]. The taxa associated with the immunocompetent survivors again include *Prevotella* and *Lactobacillus* spp. Although *Lactobacillus* spp. are typically found in the gastrointestinal tract, they have been shown to play a beneficial role in regulating respiratory immunity [57].

For week 3 and above, SARS-CoV-2, *M. hominis*, *C. segmentosum*, *Acinetobacter baumannii*, and *Lacticaseibacillus rhamnosus* were all enriched in the deceased immunosuppressed individuals. Among the taxa observed in the immunocompetent survivor group are typical airway microbes, including *Prevotella oris*, and several *Streptococcus* spp.. No taxa were significantly associated with the immunosuppressed survivor group, but some rare taxa were linked to the deceased immunocompetent group. The only taxon detected across multiple weeks was *Ureaplasma urealyticum*, which was enriched in the immunocompetent group across all 3 weeks (Fig. 2A), but in the deceased only in weeks 1 and 2 (Supplementary Fig. S6B). Although *U. urealyticum* is primarily associated with the urogenital tract, it has also been identified in upper respiratory tract infections. Both *U. urealyticum* and *M. hominis* have been reported in the respiratory tracts of lung transplant recipients experiencing airway complications [58].

### Mupirocin resistance is associated with immunosuppression

**Fig. 3:**
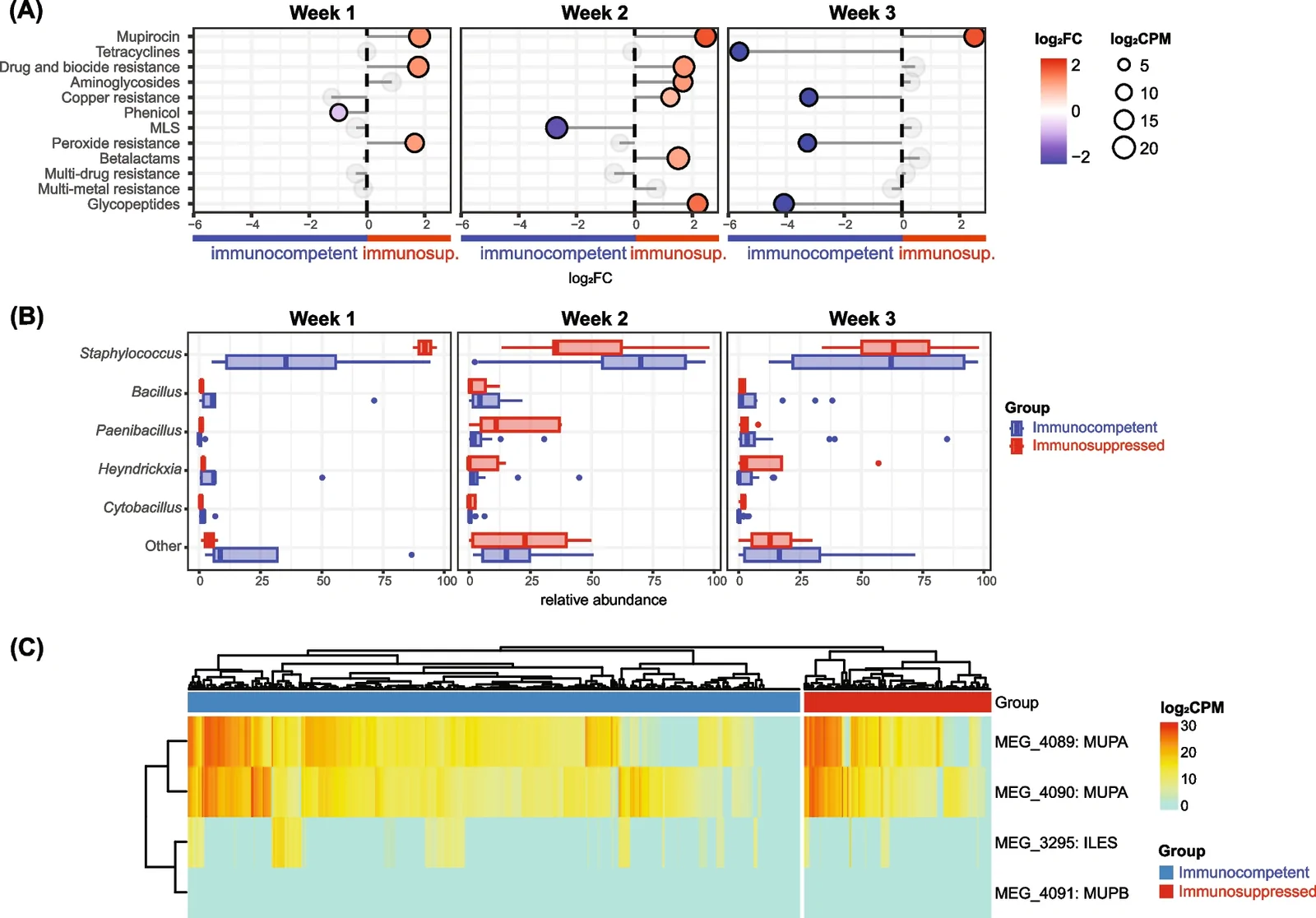
Differential expression analysis of antimicrobial resistance. Lower airway samples were stratified into 1, 2, or ≥ 3 weeks post-intubation, and differential expression analyses were performed based on clinical outcome and immunosuppression status. **A** Fold-change bubble plots showing differentially abundant antimicrobial resistance categories in the lower airways by immunosuppression status; no categories were associated with clinical outcome. Significant terms (FDR < 0.05) are shown, with color indicating enrichment (red, immunosuppressed; blue, immunocompetent) and size reflecting average log_2_CPM. **B** Reads mapping to mupirocin resistance genes were assigned to taxa using Kraken2. Reads confidently assigned at the genus level were quantified, and the relative abundance of the top contributing genera is shown. Boxes outlined in red represent samples from the immunosuppressed group, and blue outlines represent samples from the immunocompetent group. **C** Heatmap of log_2_CPM values for mupirocin resistance genes from the MEGARes database across all samples. Samples are grouped by immunosuppression status and hierarchically clustered

Since there were dynamic changes in the microbiota associated with outcome and immunocompetence status, we aimed to assess whether these changes could also be detected in antibiotic resistance markers. We conducted a differential abundance analysis of antimicrobial resistance genes between clinical outcomes and immunosuppression identified in the LA by quantifying each sample against the MEGARes antimicrobial resistance database [28]. While no categories were significantly associated with clinical outcomes, we found several differences comparing the immunosuppressed and the immunocompetent. Most notably, we identified mupirocin resistance as enriched across all ≥ 3 weeks in the immunosuppressed (Fig. 3A). Mupirocin is a topical antibiotic used to treat local skin and tissue infections, and is known to be highly effective against *Staphylococcus* spp., *Streptococcus* spp., and other Gram-negative bacteria, including *Haemophilus influenzae* [59]. In our cohort, only 68 individuals were administered topical mupirocin as a result of active surveillance protocols in place at NYU Langone. Even when controlling for mupirocin use by including mupirocin treatment as a covariate, we found mupirocin resistance enriched across all 3 weeks in the immunosuppressed group (Supplementary Fig. S8). This demonstrates that the prevalence of mupirocin resistance in immunosuppressed individuals is not a byproduct of their exposure to topical mupirocin, but rather of immunosuppression. While we detected no significant differences in the taxa contributing to mupirocin resistance between the immunosuppressed and immunocompetent groups, most mupirocin resistance sequence reads mapped to *Staphylococcus* spp. (Fig. 3B). We also identified four genes that could contribute to mupirocin resistance and found that most sequences were from two different forms of *mupA*, a gene that encodes an isoleucyl-tRNA synthetase that confers mupirocin resistance (Fig. 3C) [60]. Although mupirocin is not used to treat infections and is typically used as an anti-infective ointment, the enrichment of mupirocin resistance genes in the immunosuppressed could reflect a broader ecological shift in the LA microbiome. This enrichment could serve as an indirect marker of an increased risk of opportunistic infections, possibly exacerbated by the effects of SARS-CoV-2 infection in the LA.

### Decreased stability of microbial communities in the lower airways is correlated with immunosuppression

The enrichment of typical airway microbes in our immunocompetent and survivor groups, and the apparent loss in the immunosuppressed and deceased groups, led us to assess microbiome stability. We first examined sample stability by comparing Bray–Curtis dissimilarities between samples collected from the same individual in the same week. Comparing these Bray–Curtis dissimilarity values between the immunocompetent and immunocompromised groups, or between the alive and deceased groups, did not yield a significant difference.

Since within-patient sample dissimilarity did not capture differences between groups, we next examined stability at the community level using a comparative network analysis approach. Using the top 100 taxa ranked by median relative abundance across the LA samples, we identified the top 100 taxa at each weekly timepoint and took the union across weeks for the network analyses. In the LA (BAL), this yielded 115 total taxa, of which 83 (72%) appeared in the top 100 in all 3 weeks, 19 (17%) in 2 of the 3 weeks, and 13 (11%) in only 1 week. In the UA, the union comprised 136 total taxa, of which 64 (47%) appeared in the top 100 across all 3 weeks, 36 (26%) in 2 weeks, and 36 (26%) in only 1 week. The higher consistency observed in the LA compared to the UA suggests a more stable core microbial community in the lower airways across time, which is consistent with our broader findings.

For each week, we generated association networks for the immunocompetent and immunosuppressed groups and measured the network stability by sequentially removing the most important nodes based on degree (the number of direct connections a node has to other nodes), betweenness (how often a node lies on the shortest paths connecting other nodes, thus serving as a bridge between subnetworks), or random selection (removing nodes chosen without regard to their network position, to serve as a baseline). Stability was evaluated as the largest remaining network of interconnected nodes after each removal. These stability values were plotted and area under the curve (AUC) values were calculated, with higher AUC indicating greater network stability.

**Fig. 4:**
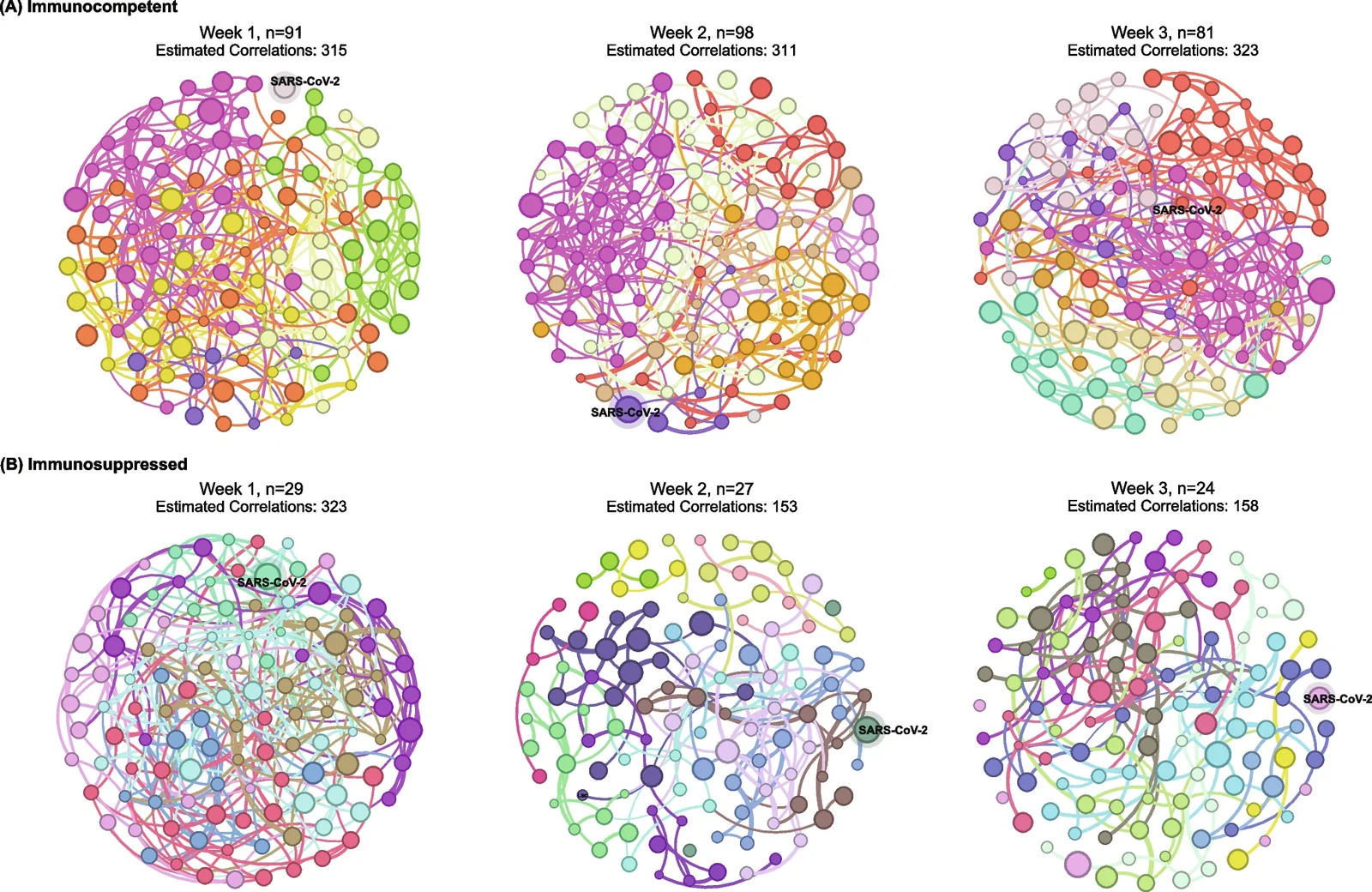
Differential networks of most abundant taxa and network stability analyses by immunosuppression status. Networks were generated using centralized log ratio (clr)-normalized counts for the top 100 most abundant taxa across all lower airway samples for each week. Comparative networks were constructed to assess taxa in **A** immunocompetent and **B** immunosuppressed groups. Nodes represent taxa and are sized by relative abundance. Edges indicate inferred associations, with thickness proportional to strength. Nodes are colored by unsupervised module assignment and SARS-CoV-2 is labeled

**Fig. 5:**
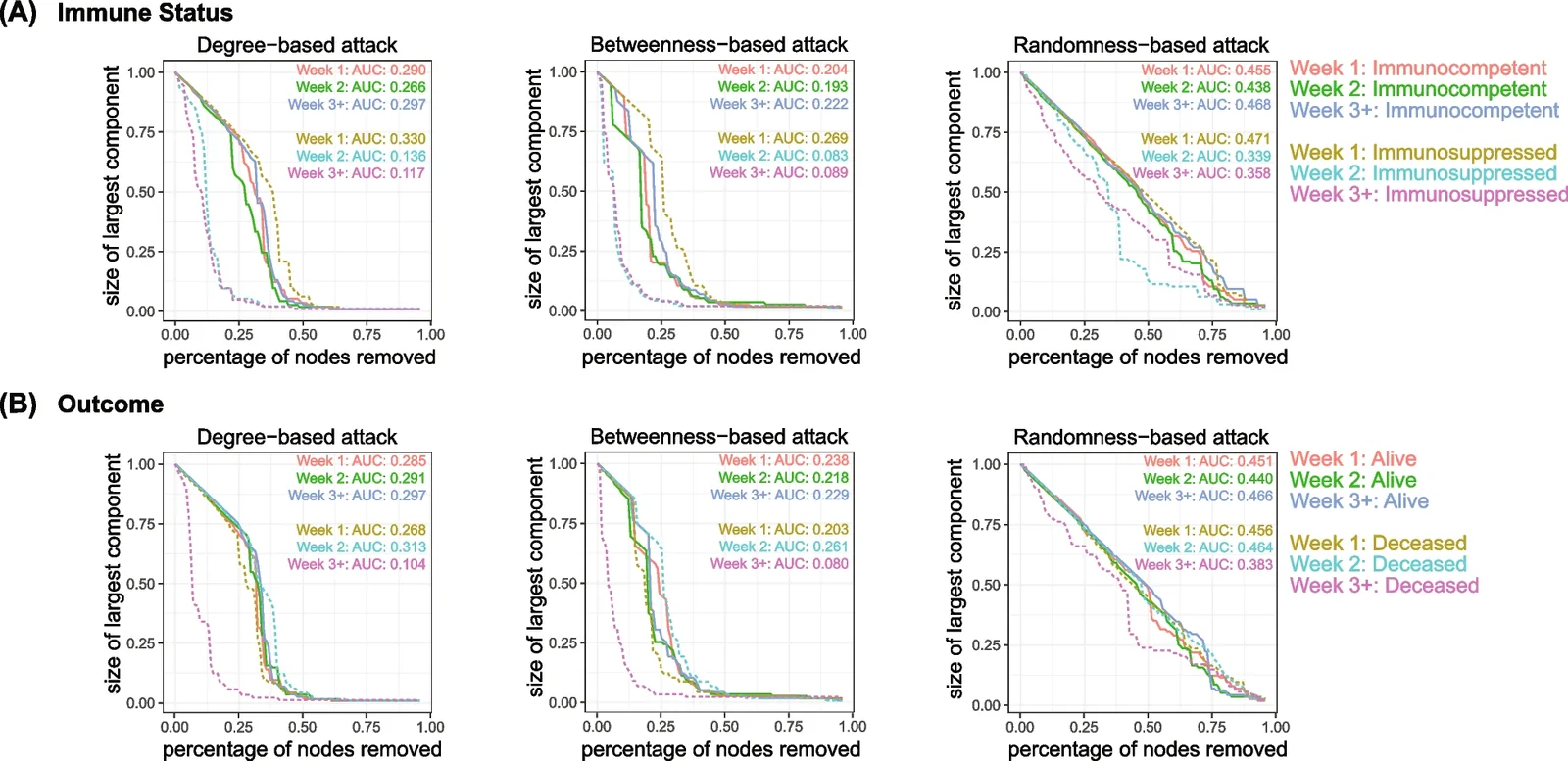
Attack-based network stability analyses for networks stratified by **A** immunosuppression status and **B** clinical outcome. Network stability was assessed by sequential removal of nodes based on degree, betweenness, or random selection. Stability was quantified as the size of the largest connected component remaining after each node removal step

Starting at week 1, the number and strength of connections between nodes in the immunocompetent (Fig. 4A) and immunosuppressed (Fig. 4B) networks are similar. However, by weeks 2 and ≥ 3, the links in the immunosuppressed network become sparser than those in the immunocompetent network. Additionally, stability remains similar throughout all 3 weeks and above in the immunocompetent (Fig. 5A), while in the immunosuppressed, network stability markedly decreases starting in week 2 (Fig. 5A). We performed the same analysis using clinical outcomes as groups for our differential networks (Supplementary Fig. S9). Using the same attack-based methods, our network stability remains fairly consistent across weeks 1–≥ 3 for the survivor group (Fig. 5B). For the deceased group, network stability shows a significant difference only at week 3 and beyond.

To address the difference in sample sizes between the immunocompetent and immunosuppressed groups, or between the alive and deceased groups, we performed network comparison analyses on 10 permuted subnetworks by randomly selecting 20% of samples from each group. For comparisons based on immunocompetence status, the AUC is significantly lower in the immunosuppressed group across all 3 weeks compared to the immunocompetent (*p* < 0.05) (Supplementary Fig. S10A). Clinical outcome comparisons show similar network stability at week 1, but by weeks 2 and ≥ 3, the AUC was significantly lower in the deceased compared to the alive (*p* < 0.05) (Supplementary Fig. S10B). This further underscores the impact of immunosuppression on the stability of the lower airway microbiome.

## Discussion

We assessed the airway microbiome of mechanically ventilated individuals within the first 2 years of COVID-19 cases in New York City. Our analyses reveal a clear distinction in the structure and dynamics of the microbiome within the lower airways, including the stability of the microbial community, between the immunocompetent and immunosuppressed along with individuals who recovered and those who died. Over more than 3 weeks of sampling, only SARS-CoV-2 was consistently associated with the deceased group and the immunosuppressed. *M. hominae* and *N. glabratus*, both associated with airway infections [58], were also found to be enriched in both groups, albeit at varying weeks across the study. Similarly, our longitudinal forecasting links an increase in both SARS-CoV-2 and these infection-linked microbes to the deceased.

In both the recovered and immunocompetent groups, we observed enrichment of typical airway microbes, including *Prevotella* spp. and *Streptococcus* spp., suggesting their contribution to the stability of the LA microbiome. Additionally, the LA microbiomes of the immunocompetent group showed between 2- to 5-fold more differentially abundant taxa than their immunosuppressed counterparts for each week. From an ecological perspective, the decrease in core airway commensals potentially reflects a breakdown in microbiome stability. The depletion of key taxa supports a link between commensal depletion and decreased network resilience in the critically ill. Network analyses of the most abundant taxa revealed decreased microbiome stability over time in the immunosuppressed and deceased groups compared to the immunocompetent and alive groups. In week 2, the network for the immunosuppressed group showed a marked decrease in stability compared to the network of the deceased group, suggesting that immunosuppression may be an earlier driver of LA microbiome instability compared to factors leading to COVID-19 mortality.

A notable observation from our host transcriptomic analysis was the prominence of mitochondrial genes among the top features predicting mortality in the BAL classifier (Supplemental Fig. S2D). Specifically, mitochondrial ribosomal RNAs, NADH dehydrogenase subunits, and cytochrome c oxidase subunits were all strongly associated with the deceased group. Rather than indicating a technical artifact, we interpret this signal as reflecting increased cellular damage and death in the airways of patients with fatal outcomes. In single-cell transcriptomics, a high proportion of mitochondrial transcripts is a well-established marker of cell stress and membrane compromise [61, 62]. In bulk BAL RNA-seq, the same principle applies at the tissue level: as airway epithelial cells, macrophages, and neutrophils undergo necrosis, their mitochondrial contents, including abundant rRNAs and mRNAs encoding oxidative phosphorylation machinery, are released into the airway lumen. This interpretation is further supported by studies showing that plasma mitochondrial DNA levels are elevated in patients with ARDS and correlate with disease severity and mortality [63, 64]. Additionally, SARS-CoV-2 has been shown to directly impair mitochondrial function through viral proteins such as ORF9b, which targets the mitochondrial outer membrane translocase TOM70 and disrupts mitochondrial protein biogenesis [65], and NSP4, which induces pro-inflammatory mitochondrial DNA release [66]. Importantly, the dominance of mitochondrial features in the classifier does not indicate overfitting: the DB-based model consistently outperformed random forest classifiers on held-out patients across all three sample types (Supplementary Fig. S2C), and the mitochondrial signal was restricted to the BAL host transcriptome—the sample type most directly reflecting airway tissue injury—while the blood transcriptome and BAL microbiome classifiers identified distinct, biologically coherent feature sets.

Our study has limitations. Although we managed to reduce the uneven and sparse sampling in our cohort caused by patient availability using DVAE forecasting, our sampling still does not represent the entire patient population. Our dataset does not capture longitudinal dynamics of mechanically ventilated patients who died early, individuals with less severe cases of COVID-19, or patients with such severe conditions (e.g., high fraction of inspired oxygen (FiO2), and positive end-expiratory pressure (PEEP)) that sampling the LA would have been even more difficult, especially via bronchoscopy. It is also possible that some of the observations could be influenced by survivor bias, which is unavoidable in investigations of critically ill patients. The potential effect of survivor bias is in part reduced by our analyses on repeated samples where earlier timepoints (week 1) are less likely to be influenced by this. In addition, it is important to note that length of stay was similar between the two groups (median[IQR] = 40[23.8–52.8] vs. 43[29.0-64.0] for the immunosuppressed and the immunocompetent groups (*p* = 0.15), respectively, indicating it is unlikely that survivor bias could influence the observations made when we compare the immunosuppressed and immunocompetent groups.

Additionally, immunosuppression can result from many different factors, and while stratifying our analyses by immunosuppression subtype would be ideal, the limited sample size within each subgroup in our cohort prevents a statistically robust comparison. Furthermore, as specimens were collected as surplus from clinically indicated procedures rather than on a fixed research schedule, our dataset likely overrepresents timepoints associated with clinical deterioration or suspected infection. This limits our ability to characterize what microbiome recovery looks like in patients who improved, and future studies incorporating scheduled longitudinal sampling, including during clinical recovery, would provide important context for the stability dynamics we describe. Our study also combined BAL and TA samples to represent the lower airways, which while empirically supported by the clustering of both sample types following sequencing run batch correction (Supplementary Fig. S1C), meant that sample type could not be fully modeled as an independent covariate due to its overlap with sequencing batch, as BAL samples were collected predominantly in earlier timepoints while TA sampling became increasingly prevalent later in the study.

Finally, although the microbiome differences we observe between immunosuppressed and immunocompetent patients suggest heightened vulnerability to opportunistic infections, we were unable to detect significant associations between ventilator-associated pneumonia (VAP) or microbiological culture results and immunosuppression status or clinical outcome. This is likely attributable to the near-universal antibiotic exposure in this cohort, which would have suppressed clinical infection signals and reduced the sensitivity of culture-based diagnostics to reflect the dysbiosis captured by metagenomic sequencing.

## Conclusions

Our findings indicate that reduced stability and a decrease in typical airway microbes in the LA microbiome are hallmarks of immunosuppression in COVID-19 patients. This raises the question of whether this pattern can explain their increased vulnerability to COVID-19 and other respiratory infections, leading to worse clinical outcomes. It also supports adopting an aggressive surveillance strategy and a low threshold for treating potential opportunistic infections among immunosuppressed patients, although the effectiveness of this approach needs to be evaluated in future prospective studies.

## Supplementary Information

Supplementary Material 1: Table S1A: Cohort metadata.

Supplementary Material 2: Figure S1.

Supplementary Material 3: Figure S2.

Supplementary Material 4: Figure S3.

Supplementary Material 5: Figure S4.

Supplementary Material 6: Figure S5.

Supplementary Material 7: Figure S6.

Supplementary Material 8: Figure S7.

Supplementary Material 9: Figure S8.

Supplementary Material 10: Figure S9.

Supplementary Material 11: Figure S10.

Supplementary Material 12: Supplementary Data.

## Data Availability

All sequencing data used for this analysis are available in the NCBI Sequence Read Archive under project numbers PRJNA688510 and PRJNA1261932. Code used for the analyses presented in the current manuscript along with data generated for the DVAE and logistic regression analysis is available at https://github.com/GhedinSGS/NYU_SARS-CoV2_immunosuppressed. This work utilized the computational resources of the NIAID HPC Skyline cluster (https://hpc.nih.gov).

## Abbreviations

ARDS: Acute respiratory distress syndrome
AUC: Area under curve
BAL: Bronchoalveolar lavage
BKG: Background
BMI: Body mass index
clr: centralized log ratio
COVID-19: Coronavirus disease of 2019
DB: Discriminative balance
DVAE: Dynamic variational auto-encoders
ELBO: Evidence lower bounds
ICU: Intensive care unit
IRB: Institutional review board
LA: Lower airway
logCPM: Log counts-per-million
logFC: Log fold-change
NYU: New York University
PERMANOVA: Permutational multivariate analysis of variance
PCA: Principal component analysis
PCoA: Principal coordinates analysis
RF: Random forest
ROC: Receiver operating characteristic
SNP: Single nucleotide polymorphism
TA: Tracheal aspirate
TMM: Trimmed mean of m-values
UA: Upper airway
VAE: Variational auto-encoders
VAP: Ventilator-associated pneumonia
