# Supplementary material for: Immunosuppression is associated with loss of airway microbiome resilience in severe COVID-19
Source: Microbiome. 2026 Jul 23;14:234. doi: 10.1186/s40168-026-02462-1 (PMC13679178; doi:10.1186/s40168-026-02462-1)

Supplementary Figure 9

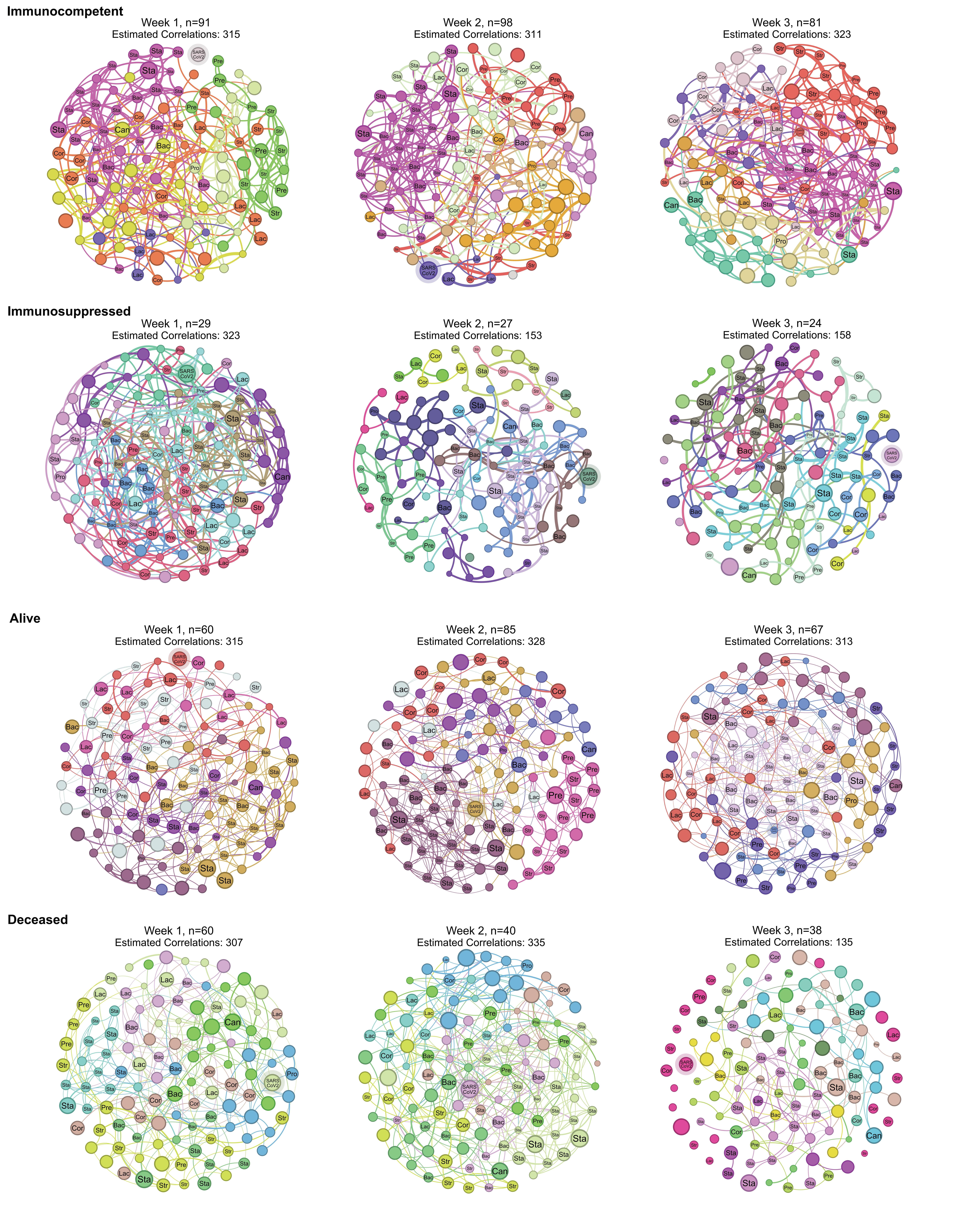

Supplement: Supplementary file 1 [file 40168_2026_2462_MOESM10_ESM.pdf]
