# Supplementary material for: Immunosuppression is associated with loss of airway microbiome resilience in severe COVID-19
Source: Microbiome. 2026 Jul 23;14:234. doi: 10.1186/s40168-026-02462-1 (PMC13679178; doi:10.1186/s40168-026-02462-1)
Supplement: Supplementary file 2 [file 40168_2026_2462_MOESM12_ESM.docx]

**ONLINE DATA SUPPLEMENT**

**Immunosuppression is associated with loss of airway microbiome resilience in severe COVID-19**

*Matthew Chung^1^, Clea R. Barnett^2,3^, Lars Hunger^4^, Imran Sulaiman^2,3^, Benjamin G. Wu^2,3,5^, Yonghua Li^2,3^, Jun-Chieh J. Tsay^2,3,5^, Shari B. Brosnahan^2,3^, Cecilia J. Chung^3^, Luis Angel^2,3^, Gregg J. Silverman^6^, James T. Morton^4^, Leopoldo N. Segal^2,3,5,7^, Elodie Ghedin^1^*^*^

^1^Systems Genomics Section, Laboratory of Parasitic Diseases, National Institute of Allergy and Infectious Diseases, National Institutes of Health, Bethesda, MD

^2^Division of Pulmonary and Critical Care Medicine, New York University Grossman School of Medicine, NYU Langone Health, New York, NY

^3^Department of Medicine, New York University Grossman School of Medicine, NYU Langone Health, New York, NY

^4^Gutz Analytics, Boulder, CO

^5^Division of Pulmonary and Critical Care Medicine, VA New York Harbor Healthcare System, NY

^6^Division of Rheumatology, Department of Medicine, New York University Grossman School of Medicine, NYU Langone Health, New York, NY

^7^Laura and Isaac Perlmutter Cancer Center, New York University Grossman School of Medicine, NYU Langone Health, New York, NY

*Corresponding Author: Elodie Ghedin, Ph.D., ([elodie.ghedin@nih.gov](mailto:elodie.ghedin@nih.gov))

# This online supplement contains 3 sections:

# 1. Supplementary Methods

# 2. Supplementary Results

3. **Supplementary Figure legends**

# 1. Supplementary Methods

## DVAE model architecture

Our DVAE model can integrate multiple omics across cohorts and sampling intervals by leveraging variational autoencoders (VAEs) in tandem with a differentiable Kalman Filter^1^. We perform Kalman Filtering in the lower-dimensional latent space, which allows us to approximate high-dimensional dynamics. The full model is detailed as follows:

${p(x}_{tk}|z_{tk})=Multinomial(\theta(z_{t k}))$, ${p(z}_{tk}|s_{t})={Normal(C_{tk}s}_{t}, Q)$

$p(s_{t}|s_{t-1}, v_{t})={Normal(A_{t}s}_{t-1}, R)$ ${q(z}_{tk}|x_{tk})=Normal(\phi_{m}(x_{tk}), \phi_{s}(x_{tk}))$

Here $x_{t k}$ represents high-dimensional omics measurements for omics *k* (microbial abundances across |*X*| microbial species), $z_{t k}$ represents the latent representation of a measurement collected at time *t* for omics *k*, and $s_{t}$ represents the latent state at time *t*. *A* represents the transition matrix between latent states, *B* represents the perturbation, and *C* represents the projection matrix to obtain the measurements $z_{t}$ from the latent state $s_{t}$. The functions $\phi_{m}$ and $\theta$ represent the encoder and decoder neural networks from the pretrained VAEs. *Q* and *R* are both pre-specified covariance matrices and $\phi_{s}$ is the encoder neural network to estimate a diagonal covariance matrix. To extend this to multiple omics levels, $x_{t}$ can represent a concatenated vector of all the omics measurements and $z_{t}$ can represent the concatenated vector of latent embeddings, output from each per-omics VAE. To fit these models, we maximize the Evidence Lower Bound (ELBO), which is given as

$${ELBO = E}_{q(s, z|x,u)}[\frac{p(x|z) p(z|s) p(s|v)}{q(z|x) q(s|z,v)}]$$

**Fitting logistic regression model**

We first split the COVID-19 patients into 80/10/10 train/validation/test splits. We then pre-trained VAEs on each of the five omics levels (BAL microbiome, BAL host transcriptome, TA microbiome, TA host transcriptome, blood transcriptome) on the training dataset. We then fine-tuned the DVAE model to forecast all five omics levels over all the time points. Upon completion, the model provides two outputs: the compressed state representation $s_{t}$ of each patient at each time point, and the predicted abundances for each omics level across all the time points. Since predictive abundances are provided at each time point, it is possible to compute log-fold changes between deceased patients and survivors at each time point, along with their credible intervals.

Once the log-fold changes are computed on the training dataset, we can construct discriminative balances ^2^, or log-ratios, that can discriminate between the deceased and the survivors. We applied two thresholds, an upper threshold of +2 and a lower threshold of -2. Taxa with log-fold changes greater than +2 are included in the numerator $L_{n}$, and taxa with log-fold changes less than -2 are included in the denominator $L_{d}$. We can then construct balances as follows where $L_{n}$ denotes the set of taxa in numerator with log-fold change greater than 2, and $L_{d}$ denotes the set of taxa in denominator with log-fold change less than -2, and x refers to the taxa abundances for BAL sample *x*. In the text, we denote $L_{n}$ as harmful taxa and $L_{d}$ as protective taxa.

$DB(x)=\log\frac{x_{L_{n}}}{x_{L_{d}}}$

Using this discriminative balance, we fitted a logistic regression model to examine the relationship between survival status and various predictors while accounting for within-subject variability. The model was specified as follows:

$survival \sim age+bmi+sex+immunosuppressed+(days \times DB)$

The dependent variable, survival, is a binary indicator of whether the subject survived. Predictors included age (a continuous variable), body mass index (BMI, modeled as a continuous predictor), sex (treated as a categorical variable), and immunosuppression status (included as a fixed effect with custom variance components to model group-specific variability). A discriminative balance (*DB*) was included to assess its predictive effect on survival outcomes, both independently and in interaction with time since symptom onset. The interaction term captured potential time-dependent changes in the effect of the discriminative balance.

## Generating comparative networks

Networks were constructed using the SPRING method with clr as the normalization method, pseudoZ0 as the zero replacement method, and a threshold of 0.3 as the sparsification method. Network stability was evaluated by sequentially removing nodes based on their importance (betweenness, degree, or random) and measuring the size of the largest remaining connected component. Stability was further quantified by comparing the AUCs calculated from each of the network stability curves. Comparative networks were generated for each week comparing the immunosuppressed versus the immunocompetent or the deceased versus alive groups. Subnetworks were generated identically by taking 10 permutations of 20% of samples from the groups comparing immunosuppressed status or clinical outcome with a p-value<0.05 (Wilcoxon rank-sum test).

# 2. Supplementary Results

## The lower airway microbiome associates better with clinical covariates

To assess whether microbiome profiles cluster based on clinical covariates, we conducted alpha diversity analyses using Shannon diversity indices and beta diversity analyses using PCoAs based on Bray-Curtis distances to compare both the LA (**Figure 1D**, **Supplementary** **Figure 3A, Supplementary Table 1C**) and UA samples (**Supplementary** **Figure 3B, Supplementary Table 1C**) individually. For alpha diversity, LA samples separate based on clinical outcome and tracheostomy status while in the UA, samples separate by weeks post-intubation and whether an individual was placed on veno-venous extracorporeal membrane oxygenation (ECMO), a clinical intervention used in patients with severe ARDS^3^. For beta diversity, both the LA and UA samples separate (Pr(>F) < 0.05) by sample timing based on weeks post-intubation. Additionally, both LA and UA separate based on the individual’s age group (<65 and 65+), immunosuppression status, history of COVID-19 vaccination, along with whether they were placed on ECMO. However, in contrast to UA samples, LA samples also exhibited distinct clustering based on BMI, sex, tracheostomy status, and clinical outcome, suggesting that the LA microbiome profile may be a more informative predictor of survivability in ventilated patients with COVID-19.

To correct for any bias from multiple samples coming from the same individual, we repeated both analyses using subsets consisting of samples from 1, 2, or ≥3 weeks post-intubation, selecting the earliest sample for each individual in each week. For alpha diversity, clinical outcome showed significant differences in weeks 2 and 3, along with tracheostomy and ECMO history among week 1 LA samples (**Supplementary** **Figure 4A, Supplementary Table 1C**). In the UA, we only observed significant differences in week 1 for tracheostomy and ECMO history (**Supplementary** **Figure** **4B**). For beta diversity, clinical outcome and tracheostomy status were the only covariates associated with significant clustering of LA samples across all time points (**Supplementary** **Figure 5A, Supplementary Table 1C**), which was not the case for UA samples (**Supplementary Figure 5B**). When dividing samples by week, immunosuppression status, BMI, ECMO history, and sex were significantly associated with LA sample clustering in more weeks compared to UA samples. This indicates that the LA microbiome profile has greater sensitivity and informative value compared to the UA in ventilated COVID-19 patients.

# 3. Supplementary Figure Legends

## Supplementary Figure 1: Overview of longitudinal cohort and Bray-Curtis PCoA.

**(A)** Dot plot illustrating the study cohort from admission (grey squares) to discharge (blue dots) or death (red dots). Intermediate points indicate sampling times for lower airway (LA; bronchoalveolar lavage [BAL] or tracheal aspirate [TA]), upper airway (UA; nasopharyngeal swabs), and background. Dashed lines indicate days post-intubation (0, 7, 14, and 21). Lines are colored by immunosuppression status (red, immunosuppressed; grey, immunocompetent).

**(B)** Histogram of sample collection dates from March 3, 2020 to March 30, 2022, colored by sample type.

**(C)** Bray-Curtis distances were calculated between all UA, BAL, TA, and background samples. ADONIS tests were used to identify significant differences (Pr(>F) < 0.05) among the four different sample types.

## Supplementary Figure 2: Dynamics proof-of-concept forecasting multi-omics profiles.

(**A**) Overview of the dynamical variational autoencoder (DVAE) framework (adapted from ^1^). Each omics dataset is compressed into a latent space (Z), enabling inference of interactions across omics layers.

(**B**) Scatterplots of observed versus predicted abundances of the blood transcriptome, BAL host transcriptome, TA host transcriptome (*inset*), BAL microbiome, and TA microbiome (*inset*).

**(C) Receiver operating‑characteristic (ROC) curves** for held‑out patients (n = 34), showing performance of linear mixed‑effects models driven by a single discriminative balance (DB) score (bold line) compared with matched random forest (RF) models (pale line) across three sample types: BAL host transcriptome, blood transcriptome, and BAL microbiome. Models were trained on 217 patients and tuned on an independent set of 28 patients. Reported AUC values are averaged over bootstrap resampling of the 34 test subjects; the black dashed diagonal indicates chance performance.

**(D)** Log‑fold change (deceased vs. alive) for the 20 features defining each balance: the 10 most overrepresented (“harmful”) features (numerator; positive values), and the 10 most under‑represented (“protective”) features (denominator; negative values). Host gene symbols are shown.

## Supplementary Figure 3: Diversity metrics across covariates in airway samples.

**(A)** Lower airway and **(B)** upper airway samples were analyzed for Shannon diversity and Bray-Curtis distances across covariates, including weeks post-intubation, age, BMI group, COVID-19 vaccination history, tracheostomy, and ECMO status. Shannon diversity was compared using Kruskal-Wallis tests (p<0.05). Bray-Curtis distances were visualized by PCoA, with group differences assessed using PERMANOVA (Pr(>F) < 0.05). Lower airway analyses stratified by immunosuppression status and clinical outcome are shown in **Figure 1D**.

## Supplementary Figure 4: Shannon diversity of airway samples by week.

Shannon diversity indices were calculated for **(A)** lower airway samples and **(B)** upper airway samples across weeks 1, 2, and ≥3 post-intubation. Differences were assessed using Kruskal-Wallis tests (p<0.05) across immunosuppression status, age group, BMI group, COVID-19 vaccination history, clinical outcome, tracheostomy, ECMO status.

## Supplementary Figure 5: Bray-Curtis PCoA of all airway samples separated by week.

Bray-Curtis distances were calculated for **(A)** lower airway and **(B)** upper airway samples across weeks 1, 2 and ≥3 post-intubation. Group differences across covariates (immunosuppression status, age, BMI, COVID-19 vaccination history, clinical outcome, tracheostomy, ECMO) were assessed using ADONIS/PERMANOVA (Pr(>F) < 0.05). Distances were visualized using PCoA.

## Supplementary Figure 6: Differential abundance by immunosuppression status and clinical outcome.

(**A**) Lower airway samples were stratified by weeks post-intubation (1, 2, or ≥3), and differential expression analyses were performed by immunosuppression status and clinical outcome. UpSet plots show intersections of differentially enriched taxa across time points.

(**B**) Fold-change bubble plots showing taxa differentially enriched in the lower airways by clinical outcome. For each week, significant taxa (FDR <0.05) are shown (red, deceased; blue, alive). Dots color reflects log-fold change, and size reflects logCPM.

## Supplementary Figure 7: Differential abundance by immunosuppression status and clinical outcome.

**(A)** To determine whether antibiotic or steroid usage in the immunosuppression differential expression analysis, we compared the differentially abundant taxa with immunosuppression alone versus adding antibiotic exposure score (AES) or steroid usage as a confounding variable. Identified differentially expressed taxa were compared between the three groups using an UpSet plot. (**B,C**) Fold-change bubble plots showing taxa differentially enriched in the lower airways as a function of immunosuppression with AES **(B)** or steroid usage **(C)** as a confounding variable. For each week, significant taxa (FDR <0.05) are shown (red, deceased; blue, alive). Dots color reflects log-fold change, and size reflects logCPM.

## Supplementary Figure 8: Differential expression of antimicrobial resistance adjusting for mupirocin usage.

Differentially abundant antimicrobial resistance categories are shown by immunosuppression status, adjusting for mupirocin usage as a covariate. Significant terms (FDR <0.05) are displayed, with color indicating direction of enrichment (red, deceased or immunosuppressed; blue, alive or immunocompetent) and size reflecting average logCPM.

## Supplementary Figure 9: Differential networks of top abundant taxa comparing clinical outcome.

Networks were generated using centralized log ratio (clr)-normalized counts for the top 100 most abundant taxa across all lower airway samples for each week. Comparative networks were constructed to compare the dynamics of these for immunosuppressed, immunocompetent, surviving (“Alive”), and deceased patients. Nodes represent taxa and are sized by relative abundance. Edges indicate inferred associations, with thickness proportional to strength. Nodes are colored by unsupervised module assignment and labeled for key taxa, including SARS-CoV-2; opportunistic pathogens such as *Proteus mirabilis* (Pro) and *Candida albicans* (Can); and taxa from common genera, including *Bacillus* spp. (Bac), *Corynebacterium* spp. (Cor), *Lactobacillus* spp. (Lac), *Prevotella* spp. (Pre), *Staphylococcus* spp. (Sta), and *Streptococcus* spp. (Ste).

## Supplementary Figure 10: Attack-based subnetwork analyses comparing immune status and outcome.

**(A)** Attack-based network stability analyses were performed across 10 permutations of subnetworks comparing immunosuppressed and immunocompetent cohorts. Area under the curve (AUC) values were compared by week using Wilcoxon rank-sum test (*: p<0.05).

**(B)** Similar analyses were conducted for subnetworks comparing deceased and surviving cohorts. Curves represent the average across permutations. Network stability was assessed by sequential removal of nodes based on degree, betweenness, or random selection, and quantified as the size of the largest connected component after each removal. AUC values were used to summarize stability, with higher values indicating greater robustness.

# References

1	Fraccaro, M., Kamronn, S., Paquet, U., and Winther, O. in *Advances in Neural Information Processing Systems 30 (NIPS 2017).* 3601–3610.

2	Quinn, T. P. & Erb, I. Interpretable Log Contrasts for the Classification of Health Biomarkers: a New Approach to Balance Selection. *mSystems* **5** (2020). <https://doi.org/10.1128/mSystems.00230-19>

3	Tisminetzky, M., Ferreyro, B. L. & Fan, E. Extracorporeal Membrane Oxygenation in COVID-19. *Crit Care Clin* **38**, 535–552 (2022). <https://doi.org/10.1016/j.ccc.2022.01.004>
